# Relative vascular permeability and vascularity across different regions of the rat nasal mucosa: implications for nasal physiology and drug delivery

**DOI:** 10.1038/srep31732

**Published:** 2016-08-25

**Authors:** Niyanta N. Kumar, Mohan Gautam, Jeffrey J. Lochhead, Daniel J. Wolak, Vamsi Ithapu, Vikas Singh, Robert G. Thorne

**Affiliations:** 1 Pharmaceutical Sciences Division, University of Wisconsin-Madison School of Pharmacy, Room #5113, Rennebohm hall, 777 Highland avenue, Madison, WI - 53705, USA; 2 Clinical Neuroengineering Training Program, University of Wisconsin-Madison Biomedical Engineering, Engineering Centers Building, 1550 Engineering Drive, Room #2120, Madison WI - 53706, USA; 3 Department of Computer Sciences, University of Wisconsin-Madison, 5780 Medical Sciences Center, 1300 University Avenue, Madison, WI - 53706, USA; 4 Neuroscience Training Program & Center for Neuroscience, Rooms 9531 & 9533, Wisconsin Institutes for Medical Research II, 1111 Highland Ave. Madison, WI - 53705, USA; 5 Cellular and Molecular Pathology Graduate Training Program, University of Wisconsin-Madison, UW Department of Pathology and Laboratory Medicine 1685 Highland Avenue Madison, WI - 53705, USA

## Abstract

Intranasal administration provides a non-invasive drug delivery route that has been proposed to target macromolecules either to the brain via direct extracellular cranial nerve-associated pathways or to the periphery via absorption into the systemic circulation. Delivering drugs to nasal regions that have lower vascular density and/or permeability may allow more drug to access the extracellular cranial nerve-associated pathways and therefore favor delivery to the brain. However, relative vascular permeabilities of the different nasal mucosal sites have not yet been reported. Here, we determined that the relative capillary permeability to hydrophilic macromolecule tracers is significantly greater in nasal respiratory regions than in olfactory regions. Mean capillary density in the nasal mucosa was also approximately 5-fold higher in nasal respiratory regions than in olfactory regions. Applying capillary pore theory and normalization to our permeability data yielded mean pore diameter estimates ranging from 13–17 nm for the nasal respiratory vasculature compared to <10 nm for the vasculature in olfactory regions. The results suggest lymphatic drainage for CNS immune responses may be favored in olfactory regions due to relatively lower clearance to the bloodstream. Lower blood clearance may also provide a reason to target the olfactory area for drug delivery to the brain.

Intranasal delivery is a well-established route to non-invasively target therapeutics to the peripheral compartment via the systemic circulation^1^. It avoids the gastrointestinal metabolism and hepatic first-pass elimination often associated with the oral route, allowing its use with peptides and protein therapeutics that are typically degraded following oral delivery^1^. Another emerging attribute of the intranasal delivery route–its ability to potentially target small fractions of therapeutics to the brain by circumventing the blood-brain barrier and blood-CSF barriers–has begun to receive much more attention in the past decade^2^,^3^,^4^. Intranasal administration has been shown to have an advantage over other parenteral systemic administration routes for the delivery of biological macromolecules such as peptides^5^,^6^, proteins^7^,^8^,^9^, oligonucleotides^10^ and gene vectors^11^ to the brain. We have previously described how labeled proteins and other macromolecule tracers may cross the nasal epithelia via paracellular or transcellular transport to reach the underlying lamina propria of the nasal respiratory and olfactory regions, after which they may (i) be absorbed into nasal blood vessels to enter the systemic circulation, (ii) be absorbed into nasal lymphatic vessels and drain to the cervical lymph nodes, or (iii) directly access extracellular pathways (perivascular, perilymphatic or perineural) associated with the trigeminal and/or olfactory nerves to reach the brain^2^,^3^,^8^,^9^. Further widespread distribution within the brain was recently shown to involve convective transport within the perivascular spaces of cerebral blood vessels^12^. In theory, preferentially targeting a region of the nasal passage that has a lower blood vessel density (vascularity) and/or more restrictive capillary permeability characteristics (size-dependent transport across vessel walls) would help minimize delivery to the systemic circulation and thus enhance access to the cranial nerve-associated extracellular pathways leading to the brain^3^; indeed, previous work has shown that intranasal application of a vasoconstrictor can significantly increase peptide delivery to the olfactory bulbs through a reduction in the systemic absorption rate (likely mediated by maintenance of higher peptide levels in the olfactory mucosa due to decreased nasal mucosal blood flow)^13^. However, very limited information currently exists describing vascularity and relative capillary permeability for the different nasal mucosal sites, despite their obvious importance for drug delivery and disposition of intranasally applied small molecules and biologics (e.g. oligonucleotides, peptides, and proteins) as well as for better understanding of nasal physiological mechanisms (e.g. lymphatic clearance and immune responses).

The nasal mucosae consist of four types of surface epithelia (squamous, respiratory, transitional, and olfactory) along with their underlying loose connective tissue compartments (lamina propria) that contain blood vessels, lymphatic vessels, glands, and nerves^14^. Although species differences are apparent in the general architecture of the nasal passages (e.g. turbinate shape), the major difference between mammals is primarily in the relative percentage areas of the respiratory and olfactory mucosae that together occupy the vast majority of the nasal cavity (e.g. about a 50:50 olfactory:respiratory area ratio is observed in rats compared to an approximately 10:90 olfactory:respiratory area ratio in primates)^2^,^14^. A small number of previous studies have examined nasal mucosal vascular extravasation under different conditions, nearly all of which have focused on nasal leakage of Evans blue-labeled macromolecules from the plasma compartment of rats^15^,^16^,^17^. Evans blue extravasation studies have demonstrated that nasal mucosal blood vessels are fairly permeable when compared to the non-permeable blood vessels of the brain^17^ and have also provided some indication that there may be vascular permeability differences between respiratory and olfactory regions of the nasal mucosa^16^; however, a detailed, quantitative comparison of vascular properties across multiple nasal mucosal regions using a range of well-characterized vascular tracers has not yet been described. Such a comparison is necessary to reveal size-dependent permeability properties of the nasal vasculature that may critically inform and guide nasal drug delivery, e.g. to provide an explanation for which particular locations of the nasal passage might be targeted for greater brain delivery (with lower systemic absorption) and to better anticipate the disposition of larger biologics.

Most biologics are hydrophilic macromolecules surrounded by a strongly adsorbed layer of water, referred to as a hydration shell, that moves with the macromolecule^18^. Capillary ‘pore theory’^19^ suggests that passive exchange of hydrophilic molecules between the systemic circulation and tissue interstitium occurs along an osmotic or chemical gradient via microscopic pores that cover capillary walls. An established method to quantify the upper limit of vascular permeability of capillaries based on this pore theory^19^ is to systemically administer exogenous tracers of various sizes and compare accumulation of the tracers in the tissue interstitium that occurs as a result of transcapillary diffusion^20^,^21^. Transport of a hydrophilic molecule through a liquid-filled pore is expected to become increasingly ‘hindered’ or ‘restricted’ when the hydrodynamic diameter (*d*_H_) of the molecule approaches the pore diameter and analytical expressions for this behavior have been described for different pore geometries^22^. Here, we quantitatively investigated extravasation at ten different nasal mucosal sites utilizing a broad size range of different hydrophilic macromolecule tracers to assess whether significant regional differences in vascular permeability characteristics exist across and within the nasal respiratory and olfactory mucosae. We also estimated relative vascularity between the nasal respiratory and olfactory mucosae by counting putative capillaries in the lamina propria of each region. For vascular permeability measurements, we systemically administered Texas Red-labeled, fixable, hydrophilic macromolecules of increasing size to different animals, constraining the moles of fluorophore administered to facilitate comparisons between each of four tracers: Lysine-fixable Texas Red-conjugated 3 kDa dextran (TR-Dex3), lysine-fixable Texas Red-conjugated 10 kDa dextran (TR-Dex10), Texas Red-conjugated bovine serum albumin (66.5 kDa) (TR-BSA) and lysine-fixable Texas Red-conjugated 70 kDa dextran (TR-Dex70). To our knowledge, this study represents the first to examine regional differences in nasal capillary permeability to hydrophilic macromolecules of varying sizes. The findings reveal important differences in mucosal vascular properties between nasal respiratory and olfactory regions that provide guidance on the region of the nasal passage that might be targeted to maximize intranasal drug delivery to the brain and that we speculate may also have significance for physiological processes such as the efficiency of lymphatic drainage to regional lymph nodes.

## Results

### Determining the free diffusion coefficients and hydrodynamic diameters of lysine-fixable Texas Red-labeled 70 kDa Dextran and Texas Red-labeled bovine serum albumin using integrative optical imaging (IOI)

**Figure 1:**
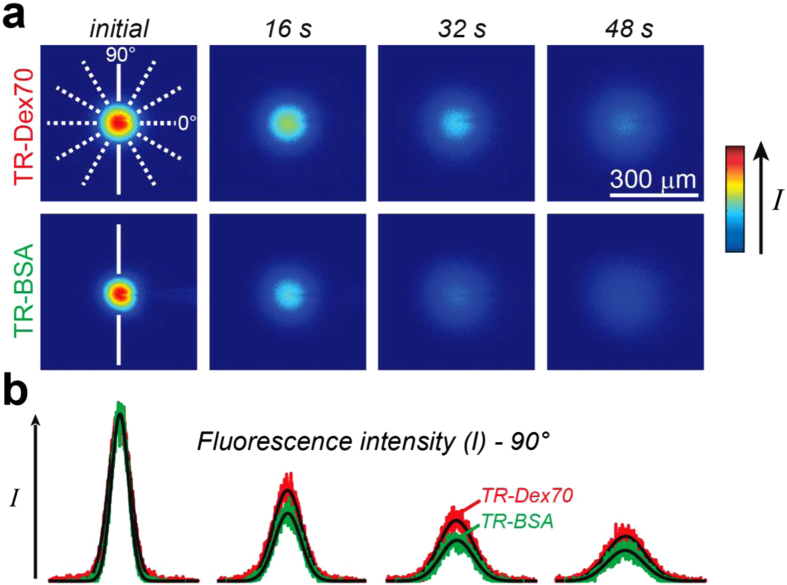
Characterization of tracer solution behavior by free diffusion measurements obtained using integrative optical imaging. (**a**) Representative images after pressure ejection of Lysine fixable texas red labeled 70 kDa dextran (TR-Dex70) or BSA (TR-BSA) into dilute agarose. (**b**) Fluorescence intensity profiles were extracted from each image (TR-Dex70 data in red; TR-BSA data in green) and fit to the diffusion equation (black lines) along one of six different axes (shown in A, solid white lines; 90° axis for TR-BSA; 90° axis for TR-Dex70). Curve fitting yielded *D* for TR-Dex70 (37 °C) = 5.0 × 10^−7^ cm^2^/second and *D* for TR-BSA (37 °C) = 8.24 × 10^−7^ cm^2^/second for the records shown.

The use of dextrans to evaluate capillary permeability to hydrophilic macromolecules has been demonstrated previously for renal glomerular^23^ and intestinal capillaries^24^. Dextrans are inert polysaccharide molecules that are well tolerated upon systemic administration in rats and mice and induce minimal vascular leakage^25^. Texas red-labeled lysine-fixable dextrans are hydrophilic and anionic at physiological pH, which results in minimal interaction with lipid plasma membranes^26^ and renders them highly water soluble, allowing the use of high concentrations and thus higher signal-to-noise during *ex vivo* fluorescence imaging^12^. The incorporation of lysine residues in the dextran conjugates also makes it possible to fix them via cross-linking using an aldehyde fixative^12^, thus eliminating the possibility of post-mortem tracer movement in tissue^12^. We recently used integrative optical imaging (IOI), a highly validated and quantitative method for measuring diffusion in different media^12^,^26^,^27^,^28^,^29^, to determine the size of Texas red-labeled 3 and 10 kDa lysine-fixable dextrans^12^ by measuring their free diffusion coefficients (*D*) and then estimating their apparent hydrodynamic diameters (*d*_H_) with the Stokes-Einstein equation^30^ (Table 1). Here, we used IOI to obtain *D* and *d*_H_ for TR-Dex70 and TR-BSA (Fig. 1, Table 1) to verify their properties and because these parameters have not yet been reported for lysine-fixable TR-Dex70, to the best of our knowledge. The *D* value we measured for TR-BSA (Table 1) agreed well with protein correlation predictions for monomeric BSA (8.6–9.2 × 10^−7^ cm^2^/s)^30^ as well as previous experimental measurements^31^. The experimental *D* and *d*_H_ for TR-Dex70 (as well as the other two dextrans) were also well in line with prior estimates using nonlysine fixable dextrans^28^ as well as correlation predictions^32^. Taken together, the results confirmed the stability and monodisperse nature of all four tracers, yielding a 2.7–12.4 nm range of tracer *d*_H_ to be used for nasal vascular permeability measurements (Table 1).

### Extravasation of systemically administered hydrophilic tracer macromolecules in the nasal mucosa corresponds inversely with tracer size and is significantly greater in nasal-associated lymphoid tissue (NALT) and respiratory regions than in olfactory regions

**Figure 2:**
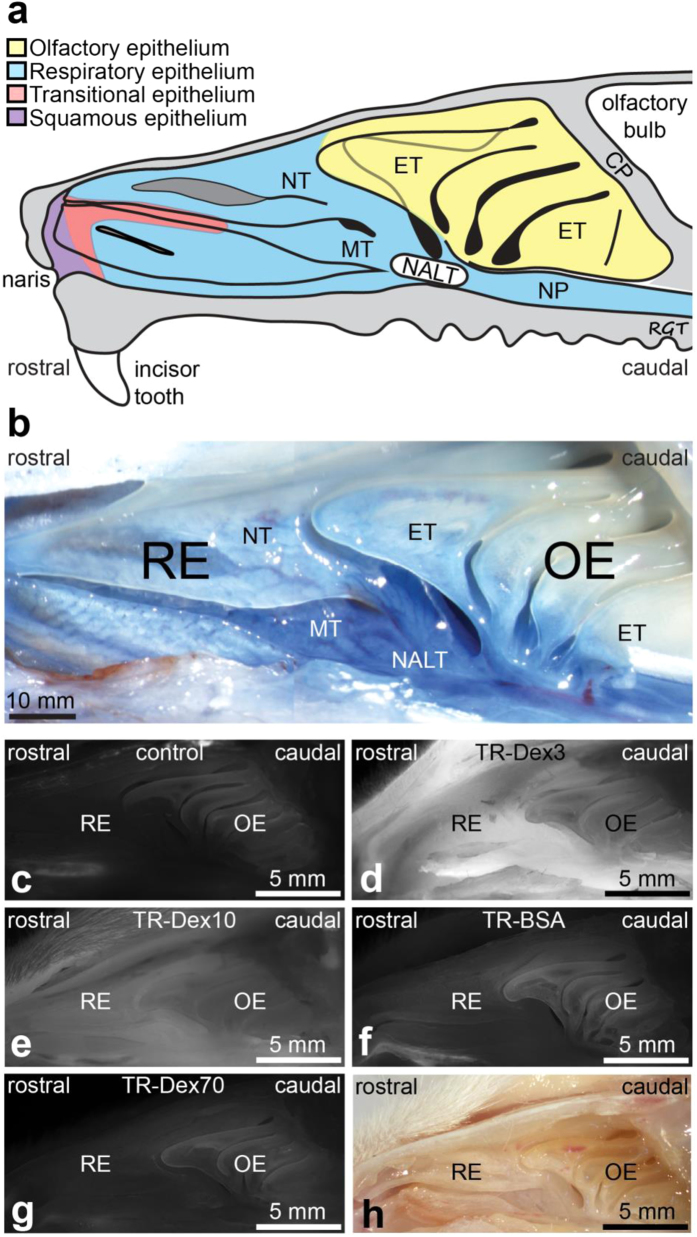
Representative images of the lateral nasal cavity wall demonstrate size-dependent tracer extravasation. (**a**) Schematic diagram showing the architecture and epithelial types lining the lateral wall of the rat nasal cavity. Abbreviations: CP, cribriform plate; ET, ethmoturbinate; NT, nasoturbinate; MT, maxilloturbinate; NP, nasopharynx; NALT, Nasal-associated lymphoid tissue (based on Mery *et al.*^33^ and Harkema *et al.*^14^). (**b**) Macroscopic aspect of the lateral wall of the rat nasal passage imaged following intra-arterial administration of 2% Evan’s blue (5 ml/kg) (based on a previous study^17^). Extravasation is noticeably greater within RE regions (NT and MT) and NALT than that observed in OE regions. (**c**) Representative image of background autofluorescence in the rat nasal cavity following intra-arterial administration of 0.9% saline (control). (**d–g**) Representative images of fluorescent tracer distribution in the rat nasal cavity following intra-arterial administration of (**d**) Texas Red-labeled lysine fixable 3 kDa dextran (TR-Dex3), (**e**) Texas Red-labeled lysine fixable 10 kDa dextran (TR-Dex10), (**f**) Texas Red-labeled bovine serum albumin (TR-BSA), and (**g**) Texas Red-labeled lysine fixable 70 kDa dextran (TR-Dex70). A bright field image of the rat nasal cavity is shown in (**h**) for comparison. Abbreviations: RE - respiratory epithelium; OE - olfactory epithelium.

Previous studies examining extravasation of Evans blue-labeled macromolecules have primarily focused on the nasal mucosa of the lateral wall^16^,^17^. We also focused our study on the lateral wall of the nasal cavity because it can be isolated intact for imaging after careful removal of the nasal septum that separates the left and right nasal chambers. Another advantage of focusing on the lateral wall is that the predominant nasal respiratory and olfactory epithelial regions lining the rat nasal cavity are distinguishable based on turbinate structure^3^,^14^,^33^ (Fig. 2a), making it an ideal location to compare observed differences in vascular permeability across these regions. The lateral wall also includes a specialized region of interest containing aggregates of nasal-associated lymphoid tissue (NALT) within the lamina propria of the mucosa, located around the opening to the nasopharynx^14^,^34^ (Fig. 2a). Brightfield imaging following intra-arterial administration and 30 min circulation of the 961 Da Evans blue dye (similar to a previous study^17^) was useful in emphasizing the boundaries between the nasal respiratory areas containing the nasoturbinate (NT), maxilloturbinate (MT), and NALT from the more caudally placed ethmoturbinates that contain the olfactory mucosa (Fig. 2b; although six different ethmoturbinates exist, only the first, third, fifth, and sixth are visible on the outer portion of the lateral wall^33^). Extravasated Evans blue in the lateral nasal wall after endpoint saline and fixative perfusion suggested greater Evans blue content in respiratory areas (NT, MT, and NALT) than in the olfactory ethmoturbinate areas, as described previously^16^,^17^. Due to the high affinity of Evans blue for the 67 kDa albumin plasma protein after its administration into the plasma compartment, regional Evans blue extravasation is commonly interpreted as positive evidence of vascular permeability to a molecule at least the size of albumin (~7 nm; Table 1)^15^,^16^,^17^, despite possible confounding by non-negligible free Evans blue and/or Evans blue binding to smaller non-albumin proteins (reviewed elsewhere^3^,^35^).

To avoid issues with Evans blue signal interpretation and facilitate comparison of relative vascular permeability across the different nasal regions with a range of highly characterized tracers of different size, we adopted a different approach based on fluorescence imaging. *In situ* extravasation signal was imaged across different animals in the lateral nasal wall following intra-arterial administration of each of the four different Texas red-labeled BSA and dextran tracers characterized above (Table 1). We constrained the administered moles of Texas Red, all experimental and post-processing steps (30 min circulation time followed by saline and fixative perfusion), and *in situ* imaging parameters across all four tracers to facilitate comparisons of size-dependent extravasation (Table 1). Control experiments utilizing intra-arterial administration of saline with experimental, post-processing, and imaging parameters identical to that in tracer-administered animals were used to determine background autofluorescence in the different nasal regions (Fig. 2c). As expected, qualitative observation confirmed that the fluorescence intensity of each extravasated tracer in the nasal mucosa corresponded inversely with tracer size, i.e. TR-Dex3 (*d*_H_ = 2.67 nm) signal (Fig. 2d) >TR-Dex10 (*d*_H_ = 4.21 nm) signal (Fig. 2e) >TR-BSA (*d*_H_ = 7.30 nm) signal (Fig. 2f) >TR-Dex70 (*d*_H_ = 12.44 nm) signal (Fig. 2g). Additionally, close inspection clearly revealed greater fluorescence signal in the respiratory NT / MT regions and NALT compared to olfactory ET regions, particularly for the two smallest tracers (TR-Dex3 and TR-Dex10).

**Figure 3:**
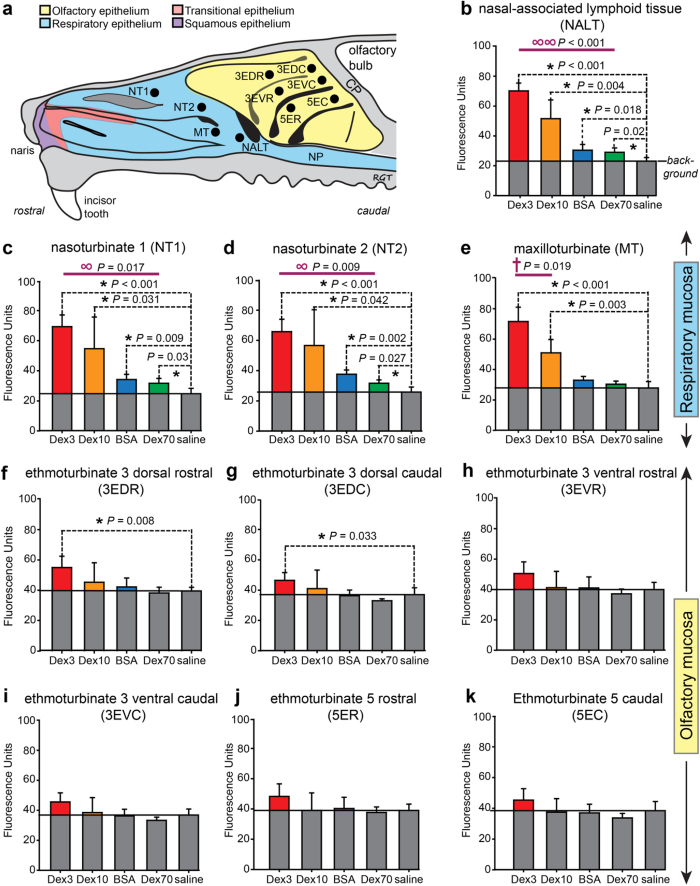
Fluorescence intensity units quantified for all tracers across various regions of the nasal mucosa. (**a**) Schematic showing regions sampled within the nasal mucosa. Abbreviations: 3ED, dorsal scroll of third ethmoturbinate (3EDR, rostral; 3EDC, caudal); 3EV, ventral scroll of third ethmoturbinate (3EVR, rostral; 3EVC, caudal); 5E, fifth ethmoturbinate (5ER, rostral; 5EC, caudal); NT1, nasoturbinate site 1; NT2, nasoturbinate site 2; MT, maxilloturbinate; NALT, nasal-associated lymphoid tissue. 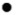 Indicate areas sampled for image analysis using ImageJ (see text and methods for further details). Fluorescence intensity (arbitrary units) within each sampled region above background for the NALT, respiratory areas (**c–e**), and olfactory areas (**f–k**) are denoted in color (i.e. grey fill denotes the level of background fluorescence for each region, determined from saline controls). Regions comprised by the respiratory mucosa and regions comprised by the olfactory mucosa are segregated to facilitate data comparisons. Values are given as means ± s.e.m. (*n* = 4) for each tracer or saline control treatment. Comparisons of data sets that followed a normal distribution used parametric statistical tests while comparisons of data sets that did not follow a normal distribution used non-parametric statistical tests. Abbreviations: ∞ One-way ANOVA (parametric) comparing fluorescence intensity above background between all tracers (α = 0.05); ∞ ∞ Kruskal-Wallis One-way analysis of variance on ranks (non-parametric) comparing fluorescence intensity above background between all tracers. † Two-tailed Student’s t-test (parametric) comparing fluorescence intensity above background between two tracers (α = 0.05). * Two-tailed Student’s t-test (parametric) comparing fluorescence intensity between each tracer and saline control (α = 0.05).

We next quantitatively analyzed the extravascular distribution of each tracer at ten distinct sites within the nasal mucosa (Fig. 3a): two different nasoturbinate sites (NT1 and NT2) and one maxilloturbinate site (MT) comprising the respiratory region; one NALT site; and six different ethmoturbinate sites (ET) from the two most accessible ethmoturbinates comprising the olfactory region. ImageJ was used to measure fluorescence intensity for each tracer in 24 pixel × 24 pixel sampling ellipses drawn around each region of interest across images obtained under identical conditions (exposure and magnification). Fluorescence intensity plots for each of the ten sites are shown with background autofluorescence levels indicated in Fig. 3b–k. Measured fluorescence intensity values were significantly above background for all four tracers only within the NALT (Fig. 3b), NT1 (Fig. 3c), and NT2 sites (Fig. 3d) of the respiratory region. The maxilloturbinate of the respiratory region yielded significant fluorescence intensity values above background only for the two smallest tracers (TR-Dex3 and TR-Dex10; Fig. 3e). Importantly, only two of the six sampled ethmoturbinate sites, representing the dorsal olfactory region of the third ethmoturbinate, exhibited fluorescence intensity values significantly above background for any tracer and this was limited to only the smallest tracer TR-Dex3 (Fig. 3f,g). Comparing across all the different sites, the value of background-subtracted TR-Dex3 fluorescence intensity was highest in the NALT, followed by the respiratory regions (NT1, NT2 and MT), with lowest levels observed in the olfactory regions (one way ANOVA; *P* < 0.001). Taken together, the results clearly show that the sampled nasal respiratory regions demonstrate significantly greater vascular permeability than the sampled olfactory regions.

### Nasal respiratory regions exhibit higher capillary density than olfactory regions

**Figure 4:**
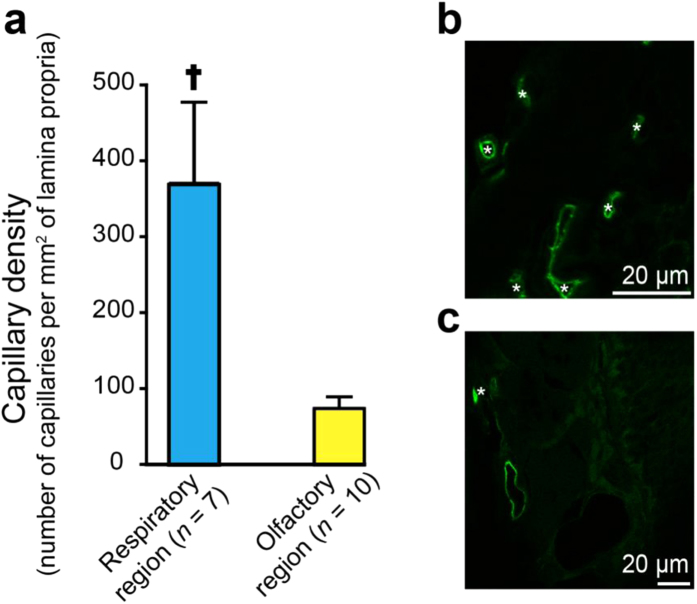
Comparison of capillary density in different regions of the nasal passage. (**a**) Measured capillary density in the respiratory region was significantly greater than the capillary density in the olfactory region. Values are given as means ± s.e.m. ^†^Mann-Whitney Rank Sum Test (non-parametric) comparing capillary density per mm^2^ of nasal lamina propria in the NALT or respiratory versus the olfactory region, *P* = 0.01. Only blood vessels with diameters less than 8 μm were counted for the analysis. Representative sections from (**b**) respiratory and (**c**) olfactory sites in the nasal mucosa were immunostained with anti- rat endothelial cell antigen (RECA-1) antibody (green). *Indicates RECA-1 positive vessels smaller than 8 μm (putative capillaries).

The primary site of tracer exchange between the systemic circulation and tissue extracellular space occurs at the level of the capillary; however, as with vascular permeability measurements, only limited information exists regarding vascularity differences between the nasal respiratory and olfactory mucosae. Yuasa has studied differences in the density of arteries and veins (excluding capillaries) between nasal regions of the Wistar rat, reporting an average vessel density of 111 large caliber (non-capillary) vessels per mm^2^ across the nasal mucosae and a relatively greater number of arteries and veins (counted together) in the olfactory mucosa compared to other areas^36^. To our knowledge, capillary density estimates between nasal mucosal regions have yet to be reported for the rat or human. We measured the mean density of capillaries (arbitrarily assigned as vessels with a lumen diameter less than 8 μm) in the respiratory (NT / MT) and olfactory (3^rd^–5^th^ET) regions of the rat nasal mucosa by counting rat endothelial cell antigen-1 (RECA-1) positive capillary profiles in randomly selected areas of the lamina propria (Fig. 4). The mean capillary density within the lamina propria of the respiratory region (*ρ*_capillary (resp.)_ = 367 ± 107 capillary profiles per mm^2^; mean ± SEM, *n* = 7) was found to be approximately 5-fold higher than that in the olfactory region (*ρ*_capillary (olf.)  _=75 ± 15 capillary profiles per mm^2^; *n* = 10) (2 tailed Student’s t-test, *P* < 0.05).

### Estimating vascular pore diameters in different regions of the nasal lamina propria using hydrodynamic theory for hindered diffusion and a normalization method

According to hydrodynamic theory for the hindered diffusion of large molecules in fluid-filled pores, tracer diffusion through pores will become increasingly hindered as the tracer hydrodynamic diameter (*d*_tracer_; here, *d*_tracer_ = *d*_H_; Table 1) approaches the pore diameter (*d*_pore_), due to hindrance arising from the pore’s limited cross-sectional area and drag from the pore walls^19^,^22^. If a relatively inert tracer’s hydrodynamic diameter is vanishingly small compared to the diameter of a vascular pore it must extravasate through (i.e. *d*_tracer_/*d*_pore_ approaches 0), the effective diffusion coefficient through the pore (*D*′) is expected to approach the tracer’s free diffusion coefficient in water (i.e. *D*′/*D* approaches 1), reflecting little if any hindrance from the pore on tracer diffusion. Conversely, if a tracer is very large such that *d*_tracer_ approaches *d*_pore_ (i.e. *d*_tracer_/*d*_pore_ approaches 1), the tracer’s effective diffusion coefficient through the vascular pore becomes much less than the free diffusion coefficient until it eventually ceases to pass (i.e. *D*′/*D* approaches 0). Conveniently, analytical expressions exist for *D*′/*D* that depend only on the *d*_tracer_/*d*_pore_ ratio and pore geometry^22^. The use and derivation of these expressions, based on hydrodynamic coefficients for axisymmetric diffusion of spheres on a centerline, have been extensively reviewed by Deen^22^. Assuming a cylindrical geometry typical of many vascular pores^19^,^37^, the following relationship applies^38^:


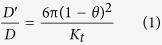


Where 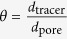
and 0≤*θ* < 1 and *K*_t_ = 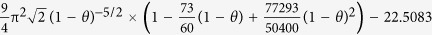 −5.6117*θ*−0.3363*θ*^2^*−*1.216*θ*^3^ + 1.647*θ*^4^

As described previously, vascular permeability for hydrophilic macromolecules can be attributed primarily to pores on the walls of capillaries^19^. Cylindrical pores in the capillary wall may be physiologically interpreted as fenestrations in the endothelium that are filled with components of the glycocalyx^39^ and residual membrane proteins^40^, or alternatively as fluid-filled channels at the interendothelial clefts bounded by a matrix of junctional proteins^41^. Theoretically, the capillary pore diameter may be obtained by measuring the extravasation of an increasingly large, relatively inert set of hydrophilic tracers across capillary pores until diffusion ceases (i.e. *D*′/*D* → 0 as *d*_tracer_/*d*_pore_ → 1). In practice, a range of well characterized tracers spanning a size range smaller than the expected capillary pore size is employed and the *relative* reduction in diffusion with increasing tracer size is interpreted according to the above-mentioned hydrodynamic theory for hindered diffusion and an assumed pore geometry (e.g. equation 1 for cylindrical vascular pores). In other words, deriving an expression for diffusive transport through constrained spaces (i.e. capillary pores) that depends on *D*′*/D* and then fitting the expression to experimental transport data for tracers of different size allows an estimate of pore size based on hydrodynamic theory.

The net transport rate describing extravasation in a particular region of the nasal lamina propria following intra-arterial administration may be expressed as follows (adapted from^42^):


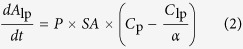


where *A*_lp_ is the amount of tracer in the interstitial fluid of the nasal lamina propria, *P* is the tracer permeability coefficient across the capillary (cm/s), *SA* is the capillary surface area in a specific region of the nasal lamina propria (cm^2^), *C*_p_ is the tracer concentration in plasma, *C*_lp_ is the tracer concentration in the nasal lamina propria, and *α* is the volume fraction of the nasal lamina propria interstitial fluid into which the systemically administered inert tracer distributes (*α* ≤ 1). At early times following intra-arterial administration of macromolecule tracers such as the dextrans and albumin employed in this study, plasma concentration will be much greater than the concentration in the nasal lamina propria (i.e. *C*_p_ ≫ *C*_lp_*/α)* allowing equation (2) to be simplified as follows:


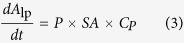


Integrating equation (3) gives the following expression, assuming *A*_lp_ = 0 at t = 0:


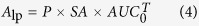


**Figure 5:**
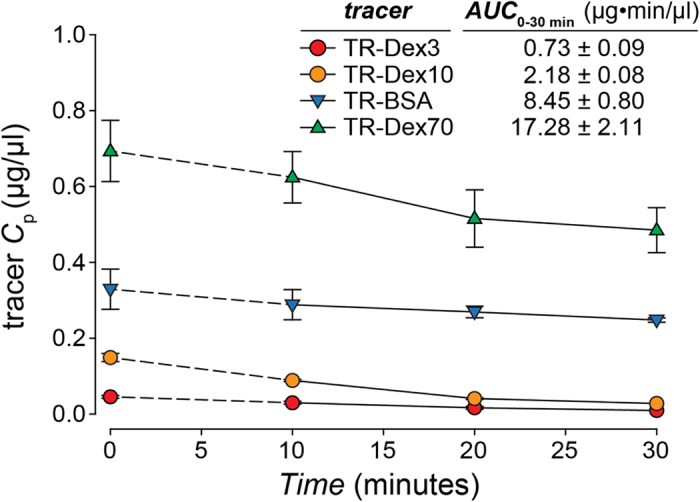
Concentration of tracer in plasma versus time. Tracer plasma concentration over time after intra-arterial (IA) administration via the abdominal aorta. Animals were administered an IA dose of Texas Red labeled fluorescent tracer at a rate of 6.25 μl/sec followed immediately by a 0.9% saline chaser bolus injection of 500 μl. Tracer dose was selected in such a way as to constrain the total moles of Texas-Red administered (Table 1). Plasma samples were acquired at 10, 20, and 30 minutes after administration of the tracer. The concentration of tracer at time zero after IA administration was estimated using a simple one compartment model (ln (*C*_p_) = ln (*C*_p_0)−(*k*_el_*t)) for all tracers.

Here, the area under the plasma tracer concentration versus time curve (*AUC*) for each tracer was measured for 30 minutes after tracer administration (i.e. *T* = 30 min), yielding 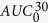 (Fig. 5; Supplementary Table S1). For our experiments, *P* can be expressed in terms of the tracer effective diffusion coefficient (*D*′) through the pore (assuming our macromolecule tracers may only access the lamina propria via transport through vascular pores) and the thickness of the capillary wall (*h*):


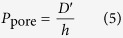


Substituting equation (5) into equation (4), we obtain:


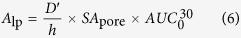


We can further rearrange equation (6) as follows:


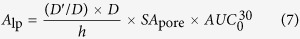


Equation (7) may be rearranged to yield an expression in terms of *D*′/*D*:


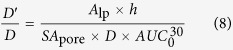


Assuming *SA*_pore_ and *h* remain constant for a given region of the nasal mucosa, equation (8) may be rewritten as a proportionality:


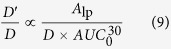


Finally, if we make the reasonable assumption that background-subtracted tracer fluorescence intensity (*FL*) for a given nasal mucosal site is a linear function of the amount of extravasated tracer in the nasal lamina propria (i.e. *A*_lp_ *α FL*), equation (9) may be rewritten as:


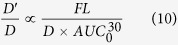


Equation (10) may be used to provide estimates of the average vascular pore diameter (*d*_pore_) in different regions of the nasal mucosae. The *D*′/*D* ratio on the left-hand side of equation (10) can be evaluated by applying our experimental *d*_tracer_ values (*d*_H_ in Table 1) and varying the value of *d*_pore_ over a range of probable values using equation (1). The right-hand side of equation (10) can be completely evaluated using our experimental data: *FL*, the arbitrary fluorescence intensity units (i.e. Texas red-associated signal) obtained for each tracer in a given region of the nasal lamina propria minus background auto-fluorescence intensity measured for the same region in saline control experiments (i.e. the colored bars in Fig. 3b–k); *D*, the free diffusion coefficient of each tracer (Table 1); and 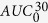, expressed in units of mol Texas red·min/L (Supplementary Table S1). Unfortunately, the constant of proportionality does not allow direct comparison between the left-hand side (*D*′/*D* ratio) and the right-hand side of equation (10) for a given tracer and nasal region. However, data from multiple tracers in a given region may be used to marginalize out the constant of proportionality by a normalization operation. In other words, normalization allows us to make use of expected behavior from hydrodynamic theory (i.e. *D*′*/D*; equation 1) and to simply vary *d*_pore_ until we find the best fit to our experimental data (i.e. the right-hand side of equation 10).

Briefly, equation (10) is of the form *X ∝ Y* and we have data for each of four tracers such that *Y*_1_, *Y*_2_, *Y*_3_, and *Y*_4_ are known while *X*_1_, *X*_2_, *X*_3_, and *X*_4_ can be evaluated explicitly by assuming a value for *d*_pore_ (i.e. from hydrodynamic theory and equation 1). Our objective was to determine the value of *d*_pore_ for a given nasal region that best fit our experimental data across multiple tracers. The method was to normalize each of the *Y*_2_, *Y*_3_, and *Y*_4_ values by *Y*_1_ to obtain the normalized *Y* terms *Y*_2_/*Y*_1_, *Y*_3_/*Y*_1_, and *Y*_4_/*Y*_1_. We then directly compared the normalized *Y* terms to similarly normalized *X* terms (*X*_2_/*X*_1_, *X*_3_/*X*_1_, and *X*_4_/*X*_1_) for a range of *d*_pore_ values. The process was repeated using each of the smaller three tracers as the normalization reference for a given nasal region (e.g. normalizing by *Y*_2_ yielded terms *Y*_1_/*Y*_2_, *Y*_3_/*Y*_2_, and *Y*_4_/*Y*_2_), although in practice normalization with data from the smaller two tracers (TR-Dex3 and TR-Dex10) yielded more robust estimates (Supplementary Figure S1). A determination of the best fit average *d*_pore_ for different nasal regions was then obtained by minimizing the mean-of-the-squared-differences (*MSD*) between the normalized *Y* terms and their corresponding normalized *X* terms (e.g. *Y*_2_/*Y*_1_ compared to *X*_2_/*X*_1_) by varying the value of *d*_pore_ over a range of probable values up to 100 nm (Supplementary Figure S2a–c; see supplementary information for details). Applying this method to our experimental data from the respiratory regions where *FL* values were significantly above background for all four tracers (nasoturbinate and NALT regions), we obtained best fit average *d*_pore_ estimates of approximately 15.0, 17.3, and 13.4 nm for the NT1, NT2, and NALT sites, respectively (Table 2). It was not possible to definitively estimate *d*_pore_ for the respiratory MT site or the six olfactory sites because only two (MT), one (3EDR and 3EDC) or none of the tracer *FL* values were significantly above background (Fig. 3e–k). However, applying the method in a limited fashion to data for the two smallest tracers from the olfactory site that exhibited the greatest extravasation of Dex3 and Dex10 (3EDR) suggested a much smaller *d*_pore_ value < 9 nm (Supplementary Figure S2d).

## Discussion

**Figure 6:**
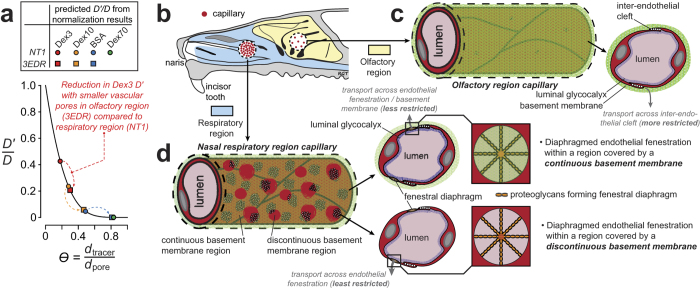
Relative vascularity and vascular permeability parameters may be used to infer physiological attributes of capillaries across different regions of the nasal mucosae. Our study demonstrated greater extravasation of systemically applied hydrophilic macromolecular tracers at NALT and nasal respiratory sites compared to olfactory sites. Extravasation was size-dependent at all sites but the differences between sites can largely be attributed to two principal physiological characteristics: (i) differences in relative capillary vascular density, i.e. vascularity, and (ii) differences in vascular permeability determined by the characteristic average capillary wall pore size^37^. (**a**) Transport of a hydrophilic molecule through a fluid-filled pore will become increasingly ‘restricted’ when the hydrodynamic diameter (*d*_H_) of the molecule approaches the pore diameter; analytical expressions for this behavior have been described for different pore geometries^22^. The plotted black line (Equation (1)) describes the relationship between *D*′/*D* for an inert tracer and θ, the ratio of the tracer hydrodynamic diameter (*d*_tracer_) to the capillary pore diameter (*d*_pore_) for a hypothetical cylindrical fluid-filled pore. Plotted symbols indicate predicted *d*_pore_ obtained from our application of hydrodynamic theory and normalization to data from the respiratory NT1 site and the olfactory 3EDR site, showing the effect of smaller pores in the olfactory site on transcapillary tracer diffusion (e.g. the TR-Dex3 effective diffusion coefficient, *D*′, is ~40% of the free diffusion coefficient, *D*, in NT1 versus only ~20% of *D* in 3EDR). (**b–d**) Schematic diagrams illustrating differences in vascularity (**b**) and hypothetical capillary pore structure in the olfactory (**c**) and nasal respiratory (**d**) regions, based on data from the present study and published findings. Our vascular permeability data and corresponding *d*_pore_ estimates suggest hydrophilic tracer transport across the capillary wall in the olfactory region likely occurs through smaller, more restrictive inter-endothelial clefts (**c**), similar to those found in skeletal muscle capillaries^41^. Hydrophilic tracer transport across the capillary wall in respiratory regions appears more consistent with diffusion across diaphragmed endothelial fenestrations (**d**), as supported by previous ultrastructural studies^37^,^49^. Transcapillary exchange will be least restrictive in capillary regions exhibiting discontinuities in the loosely packed basement membrane^49^. See text for details.

The major findings of this study are that (i) vascular permeability to hydrophilic macromolecule tracers is significantly greater in respiratory regions than in olfactory regions of the nasal passage, (ii) capillary vascularity (density) appears nearly five-fold higher in sampled nasal respiratory regions than in olfactory regions, and (iii) application of hydrodynamic theory suggests significantly larger vascular pore diameters (13–17 nm) at respiratory nasoturbinate and NALT sites compared to a rough estimate in the olfactory region (<9 nm). The reduced olfactory region extravasation we observed for all four tracers is therefore likely due to a combination of fewer capillaries (lower vascularity) and increased hindrance associated with smaller vascular pores; indeed, our estimate of pore diffusion coefficients (*D*′) from the application of hydrodynamic theory to our permeability data suggests an over 50% reduction in olfactory region *D*′ compared to the nasal respiratory region for the tracers used in this study (Fig. 6a).

Physiological interpretations of vascular permeability for hydrophilic macromolecules due to microscopic ‘pores’ on capillary walls have evolved over time. Transvascular movement of hydrophilic macromolecules is thought to occur through large pores on the capillary wall whose size is restricted by three components: the outer abluminal basement membrane^43^, the middle endothelial cell layer^44^ and the inner luminal glycocalyx^45^ (illustrated schematically for olfactory and respiratory regions in Fig. 6b–d, partly based on the results of the present study, as discussed below). Capillaries vary in their morphology with respect to the extent of these three barriers^46^, resulting in differing permeability to hydrophilic macromolecules circulating within the plasma^37^. Fenestrated capillaries of the intestinal wall, circumventricular organs, choroid plexus, endocrine glands, and skin at the extremities (e.g. fingers and soles)^37^ possess a continuous basement membrane with microdomains interrupting the plasmalemma in the form of fenestrations (generally exclusive from interendothelial clefts)^40^. A fenestration is a pore with a rim formed as a result of the plasmalemma of the luminal and abluminal sides of the endothelial cell being fused^40^,^47^. In diaphragmed fenestrated capillaries, fenestrations are occluded by a ~3 nm thick, uneven, non-lipid diaphragm^39^,^47^, composed of precipitated plasma protein^39^,^40^, glycocalyx^39^ and/or fibers of residual membrane proteins^40^; this structure is thought to restrict the passage of molecules larger than approximately 15 nm in diameter^48^. We estimated *d*_pore_ to range from ~15–17 nm in the nasoturbinate region. Ultrastructural evidence suggests the occurrence of diaphragm-covered fenestrations in the capillaries of the respiratory region and a loosely packed, sometimes discontinuous basement membrane^49^, supporting our findings (Fig. 6d). High vascularity and vascular permeability in the respiratory region may be attributed to its role in humidifying and warming inhaled air, as well as a first line of immune defense against inhaled allergens^50^. We estimated *d*_pore_ to be ~13 nm in the NALT region. High vascular permeability in NALT may be related to its immune role in facilitating antigen uptake, processing and presentation^14^,^34^. NALT, similar to the intestinal Peyer’s patches, acts as a mucosal inductive site for cell-mediated and humoral (antibody-mediated) antigen-specific immune responses. The NALT has a rich supply of lymphatic vessels that drain into the superficial and deep cervical lymph nodes, with high endothelial venules that act as sites for lymphocyte trafficking^51^.

Limited tracer extravasation within the olfactory region prevented a precise estimate of *d*_pore_. Nevertheless, our results suggest olfactory vascular pores may be much smaller (<9 nm in 3EDR) than at nasal respiratory sites, in agreement with ultrastructural observations that olfactory capillaries have few fenestrations, if any^49^, thereby limiting transport to the more restrictive interendothelial cleft as in skeletal muscle capillaries^41^. Skeletal muscle capillaries have a ~6–7 nm upper limit to permeability which is attributed to all three layers of the capillary wall^41^; however, since olfactory capillaries possess more loosely apposed endothelial cell junctions and a less compact basement membrane than that observed for skeletal muscle capillaries^48^, their clefts may be wider. Our data is consistent with that of Wolburg *et al.*^17^, who demonstrated with electron microscopy that transcardially perfused lanthanum nitrate, a small electron-dense tracer, extravasates through interendothelial clefts of olfactory blood vessels to label the subendothelial space. Although beyond the scope of the present study, it would be instructive to compare the relative expression of tight junction proteins (e.g. ZO-1, claudin-5, and occludin) between vessels of the olfactory and nasal respiratory regions; while Wolburg *et al.* have used immunocytochemistry to carefully describe ZO-1, claudin-5, and occludin positive staining of endothelial cells of the olfactory mucosa^17^, no such report yet exists for the nasal respiratory regions to the best of our knowledge. Further research will be needed to provide a better estimate of *d*_pore_ in the olfactory region as well as to examine differences in junctional proteins between different vascular regions of the nasal mucosa.

Low relative vascularity and vascular permeability within the olfactory region likely favors lymphatic drainage from the olfactory lamina propria to regional lymph nodes. Previous work has shown that proteins injected into the brain parenchyma or CSF drain along olfactory nerves to the mucosa, where they are transported to the deep cervical lymph nodes for the induction of peripheral immune responses^52^. Recent evidence shows the CNS to possess a lymphatic drainage system in the form of dural lymphatic vessels^53^; these vessels may link with trigeminal nerve components that innervate the nasal respiratory mucosa^54^ and olfactory nerve components that traverse the skull at the cribriform plate^54^. Finally, lower vascularity and vascular permeability in the olfactory region suggests intranasal targeting of drugs to that area may favor brain delivery due, in part, to reduced clearance to the systemic circulation. The present study therefore provides new evidence in support of specialized nasal spray devices^55^ that preferentially target the olfactory region when attempting to deliver intranasally applied drugs to the brain.

## Methods

### Animals

All experimental protocols were approved by the Institutional Animal Care and Use Committee at the University of Wisconsin-Madison and performed in accordance with the National Institutes of Health Guide for the Care and Use of Laboratory Animals (8th edition; 2011). Adult female Sprague-Dawley rats (180–200 g; Harlan, Indianapolis, IN, USA) were housed individually or in groups of two at room temperature under a 12-h light/dark cycle. Food and water were provided ad libitum. Animals were anesthetized with 1.5 g/kg urethane administered via intraperitoneal injections to effect. Body temperature of anesthetized animals was maintained at 37 °C using a homeothermic blanket (Harvard Apparatus). A 20 gauge cannula was then surgically inserted into the abdominal aorta.

### Tracer administration

Evans blue (MP Biomedicals, LLC) permeability experiments were essentially done as previously described^16^,^17^, allowing 30 minutes circulation time after intra-arterial dye administration (2% Evans blue in saline, 5 ml/kg), followed by saline and fixative perfusion as described below for fluorescently labeled tracers. Four different fluorescently labeled fixable tracers (Thermo Fischer Scientific) of varying sizes were administered in separate experiments to estimate the capillary pore size in the rat nasal lamina propria: Lysine fixable Texas Red-conjugated 3 kDa dextran (TR-Dex3), lysine fixable Texas Red-conjugated 10 kDa dextran (TR-Dex10), Texas Red-conjugated Bovine serum albumin (TR-BSA) and lysine fixable Texas Red-conjugated 70 kDa dextran (TR-Dex70). In order to permit comparison between the different fluorescent tracers, the total injected dose of each tracer was adjusted such that the administered number of moles of Texas Red was constant for each tracer at 5 × 10^−8^ moles of Texas red. The required amount of each tracer (Table 1) was constituted in 500 μl of 0.9% sterile saline. For all experiments, the tracer was administered intra-arterially through the abdominal aortic cannula at a rate of 6.25 μl/sec followed immediately by a 0.9% saline chaser bolus injection of 500 μl. The tracer was then allowed to circulate for 30 minutes. Tracer molecules reaching the blood vessels in the nasal passages via the systemic circulation extravasated into the lamina propria to varying degrees depending on the region in the nasal cavity and the size of the tracer. In separate experiments, blood samples were collected at 10, 20 and 30 minutes after administration in order to estimate the area under the plasma concentration versus time curve (AUC) for each tracer. At 30 minutes after tracer administration, the animals were perfused through the abdominal aortic cannula with 50 ml (~3 blood volumes) of 0.01 M phosphate buffered saline (PBS) (15 ml/min) to remove blood using a Gilson Minipuls 3 peristaltic pump. This was followed by perfusion with 500 ml fixative (4% paraformaldehyde in 0.1 M PBS; 15 ml/min). Following perfusion, each animal was immediately decapitated and the nasal mucosa carefully exposed in order to measure extravasation of the fluorescently labeled tracers into the nasal lamina propria. In order to measure any auto-fluorescence in the nasal passage, control experiments were performed similarly with intra-arterial administration of 0.9% saline instead of tracer.

### Tracer diffusion measurements and hydrodynamic size calculation

We measured the free diffusion coefficient (*D*) of TR-Dex70 and TR-BSA using integrative optical imaging (IOI), described in detail previously^12^,^26^,^27^,^28^,^29^. Briefly, a small pulse of the fluorescently labeled tracer is ejected into dilute (0.3%) agarose gel from a pulled micropipette (tip diameter = 3–6 μm) and the diffusion cloud is imaged over time using an epifluorescent microscope (Olympus BX61WI; 10x water immersion objective) connected to a CCD camera fitted with a Texas Red filter set (Fig. 1a). The short 0.1–0.2 ms, typically sub-nanoliter pulse ejection^27^ follows a point source approximation with an analytical solution to Fick’s second law that can be fit to the fluorescent intensity profiles extracted from the diffusion images (Fig. 1b). This fit is performed along six axes of the diffusion cloud and at each time point (*t*_*i*_) a parameter (*γ*_*i*_) is calculated. A linear regression of *γ*_*i*_^2^/4 versus *t*_*i*_ returns a slope equal to *D*. For each measurement, the highest and lowest *D* values are discarded, yielding an average *D* value from the remaining four axes for each individual measurement. The free diffusion coefficient of each tracer is inversely related to the hydrodynamic diameter (*d*_H_) as described by the Stokes-Einstein equation^30^:


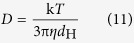


where *k* is Boltzmann’s constant, *T* is the absolute temperature (K), η is the viscosity of water (Pa*S), and *d*_H_ is the hydrodynamic diameter of the macromolecule (nm) (Table 1).

### Imaging

Fluorescence imaging of the nasal epithelium was carried out using an Olympus MVX10 fluorescent macro zoom microscope equipped with a Lumen Dynamics X-Cite 120Q illuminator, the appropriate filter set (Chroma, U-M49008XL) and a Hamamatsu C11440 Orca-flash 2.8 CMOS camera. Images of both treated and control animals were carefully acquired at the same magnification under the same light intensity and exposure time. Images were analyzed at specified regions (Figs 2 and 3a) with ImageJ using a 24 pixel x 24 pixel sampling ellipse (Rasband. http://imagej.nih.gov/ij/, 1997–2015). Brightfield imaging of the nasal epithelium was carried out using an Olympus SZ61 stereomicroscope with an Olympus DP21 camera.

### Estimating capillary density across different regions of the nasal mucosa

Animals were exsanguinated by perfusion with 50 ml 0.01 M PBS (pH 7.4) followed by fixation with 4% paraformaldehyde for 2 hours. The nasal passages were dissected out and cryoprotected overnight with 20% sucrose in PBS. The nasal passages were then flash frozen in isopentane chilled with dry ice, embedded in O.C.T. (Tissue-Tek) and cut into 15 μm sections on a cryostat (Leica CM1950). The sections were blocked with 10% goat serum in 0.01 M PBS (pH7.4) for 1 hour at room temperature. The sections were then immunostained by incubating in blocking buffer with mouse anti-Rat Endothelial Cell Antigen-1 (RECA-1) antibody (Abcam) (1:1000) overnight at 4 °C. Sections were washed in PBS and incubated for 1 hour in blocking buffer with DyLight 488 goat anti-mouse immunoglobulin G (Abcam) (1:500). After a final wash in PBS, sections were coverslipped in ProLong Diamond (Thermo Fischer Scientific). Laser scanning confocal microscopy was performed on the slices using an Olympus FV1000 confocal microscope. ImageJ was used to measure the total area of the lamina propria and count the number of capillaries (RECA-1 positive vessels with a diameter less than 8 μm) in multiple sections of the respiratory and olfactory regions to calculate the capillary density in these regions of the nasal mucosa (Fig. 4).

### Statistical analysis

Fluorescence intensity data (*FL* units) obtained using ImageJ was analyzed and graphed using SigmaPlot (version 11.2; Systat Software, Inc., San Jose, CA, USA). Least squares analysis carried out to estimate capillary pore diameter was performed using MATLAB R2014a (The MathWorks Inc., Natick, Massachusetts, United States).

## Additional Information

**How to cite this article**: Kumar, N. N. *et al.* Relative vascular permeability and vascularity across different regions of the rat nasal mucosa: implications for nasal physiology and drug delivery. *Sci. Rep.* **6**, 31732; doi: 10.1038/srep31732 (2016).

## Supplementary Material

Supplementary Information

## Figures and Tables

**Table 1:** Quantitative parameters for Texas Red-labeled tracers.

	TR-Dex3	TR-Dex10	TR-BSA	TR-Dex70
Approximate molecular weight (Da)	3000	10,000	67,000	70,000
Free diffusion coefficient (*D*, × 10^−7^ cm^2^/s) (*n*)	24.6 ± 0.20 (12)^a^	15.6 ± 0.15 (13)^a^	9.00 ± 0.19 (15)^b^	5.28 ± 0.12 (15)^b^
Hydrodynamic diameter (*d*_H_; nm)*	2.67 ± 0.02	4.21 ± 0.04	7.30 ± 0.16	12.44 ± 0.29
Moles of Texas Red fluorophore per mole of tracer	0.4	1	3	3
Intra-arterial tracer dose (μg)†	375	500	1110.6	1166.7
Intra-arterial Texas Red dose (moles)	5.0 × 10^−8^	5.0 × 10^−8^	5.0 × 10^−8^	5.0 × 10^−8^

Values reported as mean ± SEM (*n* independent measurements).

^*^Apparent hydrodynamic diameter determined from the Stokes-Einstein equation [*d*_H_ = (k*T*)/(3π*ηD*), where k is the Boltzmann’s constant, *T* is absolute temperature, *D* is the free diffusion coefficient determined from IOI (37 ± 0.5 °C), and *η* is the viscosity of water (6.9152 × 10^−4^ Pa·s at *T* = 310 K)].

^†^dissolved in saline.

^a^Lochhead *et al.*^12^.

^b^Current study. TR-Dex3, Texas Red-labeled lysine fixable 3 kDa dextran; TR-Dex10, Texas Red-labeled lysine fixable 10 kDa dextran; TR-BSA, Texas Red-bovine serum albumin; TR-Dex70, Texas Red-labeled lysine fixable 70 kDa dextran.

**Table 2:** Estimated capillary *d*_pore_ (nm) in different regions of the nasal mucosa.

Region	Normalization tracer	*d*_pore_ estimate (nm)
NT1	TR-Dex3	14.46
	TR-Dex10	13.30
	TR-BSA	17.13
	**mean ± s.e.m**	**14.96 ± 1.14**
NT2	TR-Dex3	17.16
	TR-Dex10	15.79
	TR-BSA	18.93
	**mean ± s.e.m**	**17.29 ± 0.91**
NALT	TR-Dex3	12. 61
	TR-Dex10	12.44
	TR-BSA	15.13
	**mean ± s.e.m**	**13.39 ± 0.87**

Capillary pore size was estimated by normalization with each tracer for the NT1, NT2 and NALT sites of the nasal mucosa and expressed as mean ± SEM. Abbreviations: NT1, Nasoturbinate site 1; NT2, Nasoturbinate site 2; NALT, nasal-associated lymphoid tissue. See text and supplementary information for details.
