# Supplementary material for: Relative vascular permeability and vascularity across different regions of the rat nasal mucosa: implications for nasal physiology and drug delivery
Source: Sci Rep. 2016 Aug 25;6:31732. doi: 10.1038/srep31732 (PMC4997340; doi:10.1038/srep31732)
Supplement: Supplementary Information [file srep31732-s1.pdf]

Supplementary information for:

Relative vascular permeability and vascularity across different regions of the rat nasal mucosa: implications for nasal physiology and drug delivery.

Niyanta N. Kumar[1], Mohan Gautam[1], Jeffrey J. Lochhead[1], Daniel J. Wolak[1, 2], Vamsi Ithapu[3], Vikas Singh[3] and Robert G. Thorne*[1, 2, 4, 5]

[1]Pharmaceutical Sciences Division, University of Wisconsin-Madison School of Pharmacy, [2]Clinical Neuroengineering Training Program, [3]Department of Computer Sciences, University of Wisconsin-Madison, [4]Neuroscience Training Program & Center for Neuroscience, and [5]Cellular and Molecular Pathology Graduate Training Program, University of Wisconsin-Madison, Madison, Wisconsin 53705, United States.

Contents:

Supplementary section 1. Rationale for the method used for capillary wall pore size estimation.

Supplementary section 2. Fitting the cylindrical pore model[1] to the observed fluorescence intensity data in the nasal mucosa predicts capillary wall pore size in the mucosa.

Supplementary table S1

Supplementary table S2

Supplementary figure S1

Supplementary figure S2

References

Supplementary section 1. Rationale for the method used for capillary wall pore size estimation.

An established method to quantify the upper limit of vascular permeability of capillaries for hydrophilic macromolecules based on the pore theory[2] is to systemically administer exogenous tracers of various sizes and compare accumulation of the tracers in the tissue interstitium that occurs as a result of transcapillary diffusion[3,4]. Transport of a hydrophilic molecule through a fluid-filled pore is expected to become increasingly 'hindered' or 'restricted' when the hydrodynamic diameter (d_H) of the molecule approaches the pore diameter and analytical expressions for this behavior have been described for different pore geometries[5]. Here, we quantitatively investigated extravasation of a broad size range of systemically administered hydrophilic macromolecule tracers to assess whether significant regional differences in vascular permeability characteristics exist across and within the nasal respiratory and olfactory mucosae. This experimentally observed quantitative data showing size-dependent extravasation of tracers in different regions of the nasal mucosa was then fit to a model for 'motion of a closely-fitting sphere in a fluid filled tube' (equation (1))[1] to estimate a diameter for capillary wall pores in different regions of the nasal mucosa.

Supplementary section 2. Fitting the cylindrical pore model to the observed fluorescence intensity data in the nasal mucosa predicts capillary wall pore size in the mucosa.

To obtain our experimental data reflecting relative permeability to hydrophilic macromolecules in different regions of the nasal mucosa, four fluorescent Texas Red (TR) labeled hydrophilic macromolecular tracers of increasing hydrodynamic diameters (TR-Dex3 < TR-Dex10 < TR-BSA < TR-Dex70) (Table 1) were administered systemically in separate animals. In each case, the tracer was allowed to circulate for 30 minutes following which the animal was exsanguinated by perfusion with phosphate buffered saline (PBS) to eliminate blood borne signal and fixative to prevent post-mortem movement of the extravasated tracer in the tissue interstitium. The nasal passages were then exposed and tracer extravasation into the nasal mucosa was quantified by measuring Texas Red fluorescence signal (Fig. 3). Auto-fluorescence was determined and subtracted by carrying out control experiments where saline alone was systemically administered in place of tracer. Since tracers were conjugated to different amounts of Texas Red, tracer doses were selected so as to constrain the moles of Texas Red in each experiment (Table 1).

We have previously derived the relationship:

$$\frac{D'}{D} \propto \frac{FL}{D \times AUC_0^{30}} \qquad (10)$$

where D' is the effective diffusion coefficient through a capillary wall pore, D is the free diffusion coefficient of each tracer, FL, the arbitrary fluorescence intensity units (i.e. Texas red-associated signal) obtained for each tracer in a given region of the nasal lamina propria minus background auto-fluorescence intensity measured for the same region in saline control experiments (i.e. the colored bars in Fig. 3b-k), and AUC_0^{30} is the area under the tracer plasma

concentration versus time curve measured for the 30 minute duration of the experiment. These AUC_0^{30} values for each tracer (Fig. 5) can also be expressed in units of mol Texas red·min/L of plasma (Supplementary table S1).

Briefly, equation (10) is of the form $X \propto Y$, where $X = D'/D$ and $Y = \frac{FL}{D \times AUC_0^{30}}$.

We calculated the right hand side of equation (10) i.e., the *Y* value for each tracer in different regions of the nasal mucosa using our experimental data as shown in Supplementary table S1. Y_1, Y_2, Y_3, and Y_4 are known from experimental data for different regions of the nasal mucosa, where subscript 1 corresponds to TR-Dex3, 2 corresponds to TR-Dex10, 3 corresponds to TR-BSA and 4 corresponds to TR-Dex70 (Supplementary table S1). X_1, X_2, X_3, and X_4 can be evaluated explicitly using the cylindrical pore model (equation (1)) by assuming a value for d_{pore}.

Assuming a cylindrical geometry typical of many vascular pores[2,6], the cylindrical pore model provides the following relationship[1]:

$$\frac{D'}{D} = \frac{6\pi(1-\theta)^2}{K_t} \qquad (1)$$

Where $\theta = \frac{d_{\text{tracer}}}{d_{\text{pore}}}$ and $0 \le \theta < 1$

and K_t =

$$\frac{9}{4}\pi^2\sqrt{2}(1-\theta)^{-5/2} \times \left(1 - \frac{73}{60}(1-\theta) + \frac{77293}{50400}(1-\theta)^2\right) - 22.5083 - 5.61117\theta - 0.3363\theta^2 - 1.216\theta^3 + 1.647\theta^4$$

For a specific tracer with a fixed diameter (d_{tracer}) the *X* terms (i.e., *D'*/*D*) are a function of d_{pore}. Our objective was to determine the value of *X* and thus d_{pore} for a given nasal region that best fit our experimental data (*Y* value) across multiple tracers. When evaluating *X* terms using the cylindrical pore model, the lower limit for the d_{pore} values was set to the hydrodynamic diameter (d_H) of the tracer since the cylindrical pore model is applicable only when $d_{\text{tracer}} < d_{\text{pore}}$ (equation (1)). The upper limit for d_{pore} values was arbitrarily set to 100 nm, since the largest fenestrations would be expected to have an upper limit of permeability below this value[6]. *X* values were obtained using MATLAB for all d_{pore} values within the aforementioned range with a resolution of 0.0001 nm.

The constant of proportionality in equation (10) does not allow a direct comparison between the cylindrical pore model predicted *X* terms (left hand side of equation (10)) and the experimentally measured *Y* terms (right hand side of equation (10)). However, since we have data for multiple tracers in a given nasal mucosal region, we were able to marginalize out this constant of proportionality by a normalization operation. The method was to normalize each of the Y_2, Y_3, and Y_4 values by Y_1 to obtain the normalized *Y* terms Y_2/Y_1, Y_3/Y_1, and Y_4/Y_1 (where

subscript 1 corresponds to TR-Dex3, 2 corresponds to TR-Dex10, 3 corresponds to TR-BSA and 4 corresponds to TR-Dex70). We then directly compared the normalized *Y* terms to cylindrical pore model predicted *X* terms normalized in a similar way i.e. (X_2/X_1, X_3/X_1, and X_4/X_1) for the aforementioned range of d_{pore} values. The process was repeated using each of the tracers as the normalization reference for a given nasal region (e.g. normalizing by Y_2 yielded terms Y_1/Y_2, Y_3/Y_2, and Y_4/Y_2 and so on). Experimental data (*Y*) following normalization is provided in Supplementary table S2.

Normalization of cylindrical pore model predicted *X* terms and fitting the model predicted terms to our experimental data (*Y* terms) was carried out using MATLAB. First, the squared differences between the normalized experimental (*Y*) values and corresponding normalized cylindrical pore model predicted (*X*) values over the aforementioned range of d_{pore} values was calculated. An example of squared differences across the entire range of d_{pore} values for each tracer normalization is shown for the Nasoturbinate site 1 (NT1) area of the respiratory region (Supplementary Fig. S1).

We found that normalization with data from the smaller two tracers (TR-Dex3 and TR-Dex10) yielded more robust estimates. Normalization by TR-Dex70 for all regions and additionally by TR-BSA for the third dorsal rostral ethmoturbinate (3EDR) olfactory region resulted in much larger estimated d_{pore} values (Supplementary Fig. S1d). Based on the value ranges of the predicted *X* for the four different normalizations, it was observed that the 'ranges' of the squared differences between normalized predicted (*X*) and normalized experimental (*Y*) values increased with the size of the tracer, i.e. the squared differences were smallest for TR-Dex3 and largest for TR-Dex70 normalization (see y-axis values from Supplementary Fig. S1). The very large squared difference ranges were close to the MATLAB numerical limits (Supplementary Fig. S1d) and caused the resolution of predicted d_{pore} to be poor. Hence TR-Dex70 normalization was neglected for reporting the best estimates in the respiratory region and NALT while TR-BSA and TR-Dex70 normalization was neglected for reporting the putative best estimates for the olfactory region 3EDR.

Finally, a determination of the best fit average d_{pore} for different nasal regions was obtained by minimizing the mean-of-the-squared-differences (*MSD*) between *X* and *Y* values for a particular tracer normalization in each nasal region (Supplementary Fig. S2). The final estimated capillary wall pore size for a particular region of the nasal passage was then the d_{pore} value corresponding to the average *MSD* value across all tracer normalizations (Table 2).

Supplementary table S1: Using experimentally observed fluorescence intensity data in different regions of the nasal mucosa to allow comparison to the cylindrical pore model.

Nasal region	Tracer	*FL*	*D*, × 10^{-7} cm^2/s	AUC_0^{30} (mol Texas red·min/L)	$\frac{FL}{D \times AUC_0^{30}} = Y$
NALT	TR-Dex3	47.125	24.6	9.788E-17	1.95714E+23
	TR-Dex10	28.525	15.6	2.1754E-16	8.40547E+22
	TR-BSA	7.35	9	3.81253E-16	2.14206E+22
	TR-Dex70	5.975	5.28	7.40674E-16	1.52784E+22
NT1	TR-Dex3	44.475	24.6	9.788E-17	1.84709E+23
	TR-Dex10	29.85	15.6	2.1754E-16	8.79591E+22
	TR-BSA	9.325	9	3.81253E-16	2.71765E+22
	TR-Dex70	6.825	5.28	7.40674E-16	1.74518E+22
NT2	TR-Dex3	39.925	24.6	9.788E-17	1.65812E+23
	TR-Dex10	30.8	15.6	2.1754E-16	9.07584E+22
	TR-BSA	11.75	9	3.81253E-16	3.42438E+22
	TR-Dex70	5.825	5.28	7.40674E-16	1.48948E+22
3EDR[a]	TR-Dex3	15.475	24.6	9.788E-17	6.4269E+22
	TR-Dex10	5.75	15.6	2.1754E-16	1.69435E+22
	TR-BSA	2.625	9	3.81253E-16	7.65022E+21

Table legend: Fluorescence intensity values above background autofluorescence (i.e. *FL*) (Fig. 3), the free diffusion coefficient (*D*) (Table 1), and the area under the plasma concentration versus time curve from 0 to 30 minutes for the Texas Red signal corresponding to each tracer (AUC_0^{30}) were used to calculate a term *Y.* As explained in supplementary section 2, *Y* corresponds to the right hand side of equation (10). [a]The 3rd dorsal rostral ethmoturbinate (3EDR) region had the highest extravasation of all four systemically administered tracers within the olfactory region. We therefore use the tracer fluorescence intensity above background in this area as representative of the olfactory region. In 3EDR fluorescence intensity was significantly above background only for TR-Dex3, while fluorescence intensity above background for TR-Dex10 and TR-BSA showed a trend towards significance. We therefore, provide the results for the 3EDR region as a putative estimate that is used for comparison to other regions in our discussion but exclude it from our results in Table 2.

Supplementary table S2: Normalizing experimentally observed fluorescence intensity data in different regions of the nasal mucosa to allow comparison to the cylindrical pore model.

Nasal region	Tracer	Y_z/Y_1	Y_z/Y_2	Y_z/Y_3	Y_z/Y_4
NALT	TR-Dex3	1	2.328414874	9.136719999	NA
	TR-Dex10	0.429476727	1	3.924008604	NA
	TR-BSA	0.109448467	0.254841439	1	NA
	TR-Dex70	0.078064651	0.181766895	0.713254861	NA
NT1	TR-Dex3	1	2.09993705	6.796626555	NA
	TR-Dex10	0.476204751	1	3.236585856	NA
	TR-BSA	0.147131815	0.308967549	1	NA
	TR-Dex70	0.094483196	0.198408765	0.642167001	NA
NT2	TR-Dex3	1	1.82695926	4.842095491	NA
	TR-Dex10	0.54735758	1	2.650357671	NA
	TR-BSA	0.206522156	0.377307565	1	NA
	TR-Dex70	0.089829479	0.164114799	0.434962917	NA
3EDR	TR-Dex3	1	3.793127733	NA	NA
	TR-Dex10	0.263634676	1	NA	NA
	TR-BSA	0.119034365	0.45151255	NA	NA

Table legend: As explained in supplementary section 2 the proportionality in equation (10) of the form $X \propto Y$ was eliminated by normalizing the *Y* term corresponding to each tracer (supplementary table S1) by each of the other tracers to obtain normalized *Y* values. Here subscript *z* for *Y* values corresponds to the four tracers from 1 to 4, where 1 is TR-Dex3, 2 is TR-Dex10, 3 is TR-BSA and 4 is TR-Dex70. As explained in supplementary section 2, due to poor fit of experimental data to the model, TR-Dex70 normalization was neglected for reporting the best estimates in the respiratory region and NALT while TR-BSA and TR-Dex70 normalization was neglected for reporting the putative best estimates for the olfactory region 3EDR. Neglected values are denoted as 'not applicable' or NA.

Supplementary Fig. S1: Squared differences between normalized cylindrical pore model predicted *X* values and normalized experimentally observed *Y* values.

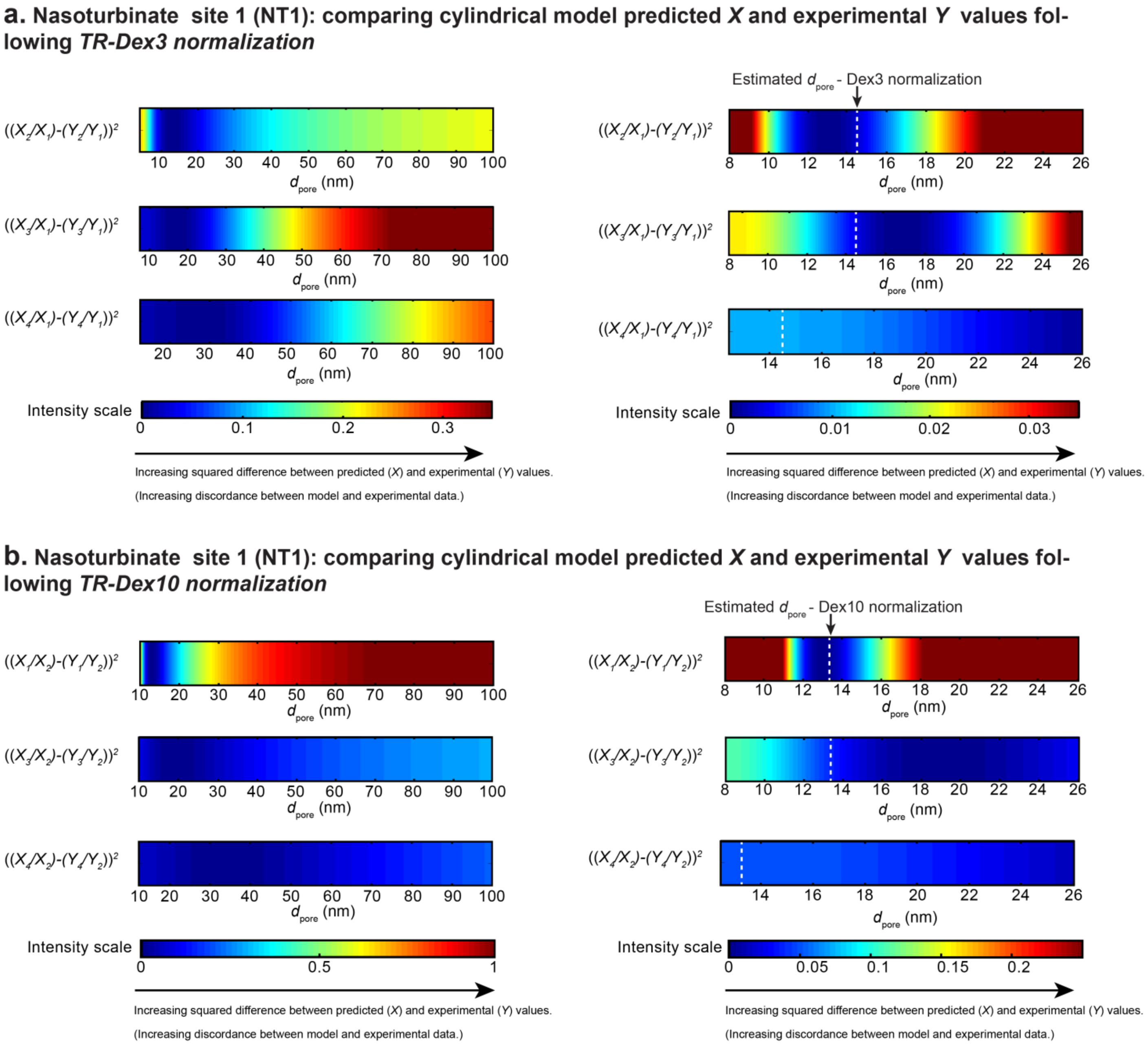

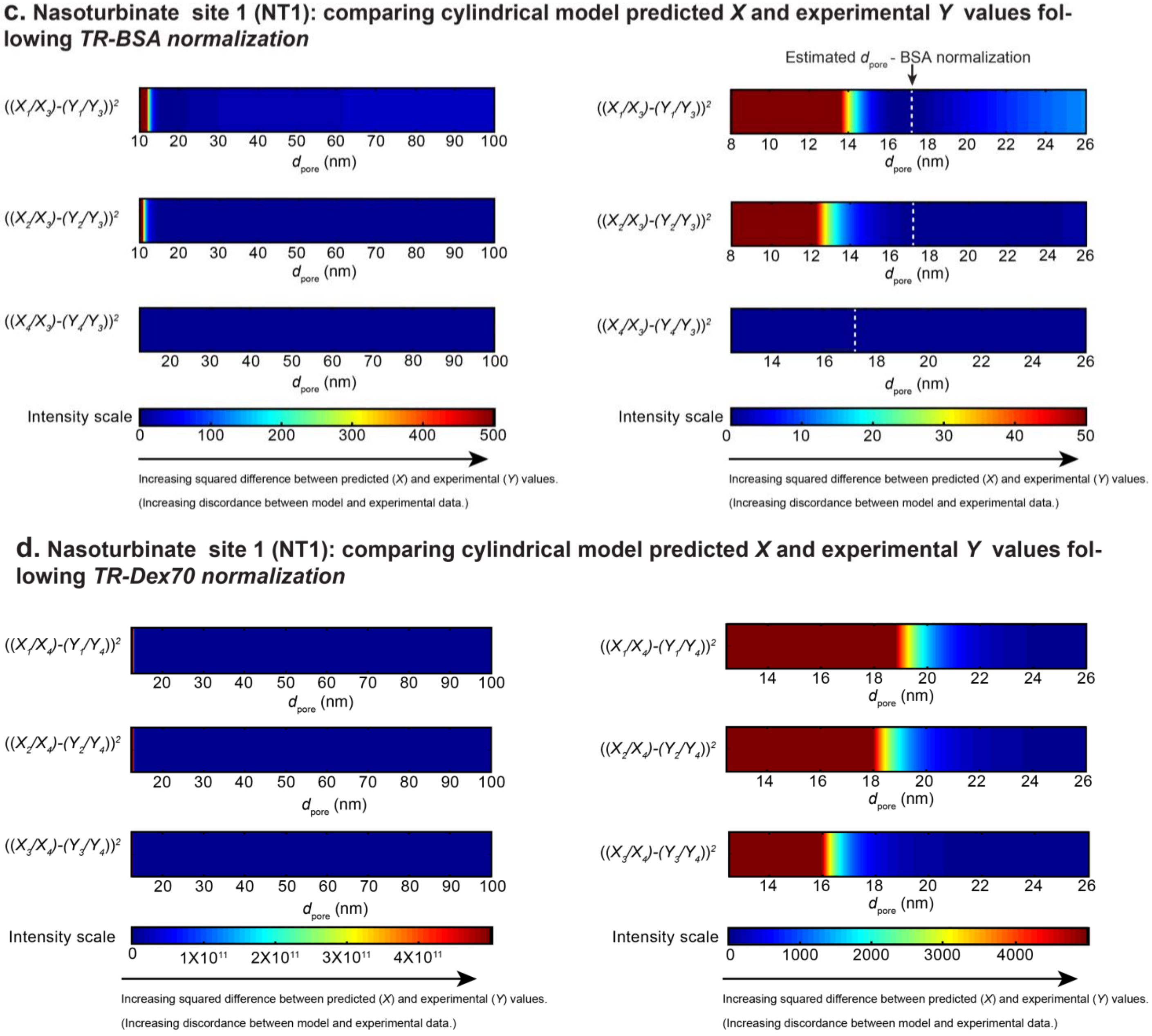

Figure Legend: Squared differences between normalized *X* and *Y* values (equation (10)) are shown for Nasoturbinate site 1 (NT1) area of the respiratory region across the entire range of d_{pore} values (left column) as well as a narrower range (right column) to indicate the final d_{pore} value determined from the mean-of-the-squared-differences (*MSD)* minimization procedure (white dashed line). Here subscript for *X* and *Y* values corresponds to the four tracers from 1 to 4, where 1 is TR-Dex3, 2 is TR-Dex10, 3 is TR-BSA and 4 is TR-Dex70.

Supplementary Fig. S2: Fitting the cylindrical pore model to the observed fluorescence intensity data in the nasal mucosa to predict capillary wall pore size in the mucosa.

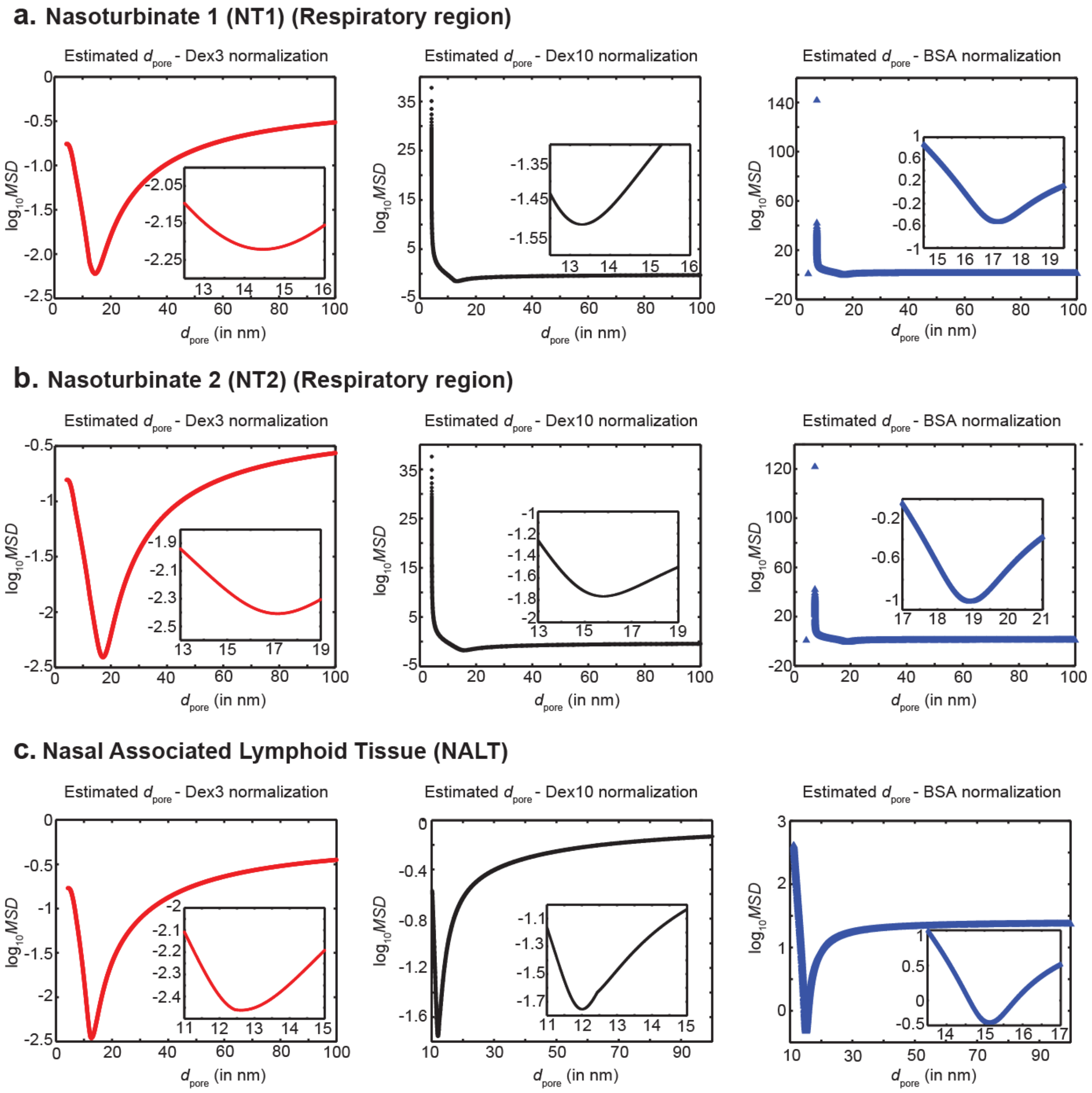

d. Rostral 3rd ethmoturbinate (3EDR) (Olfactory region)

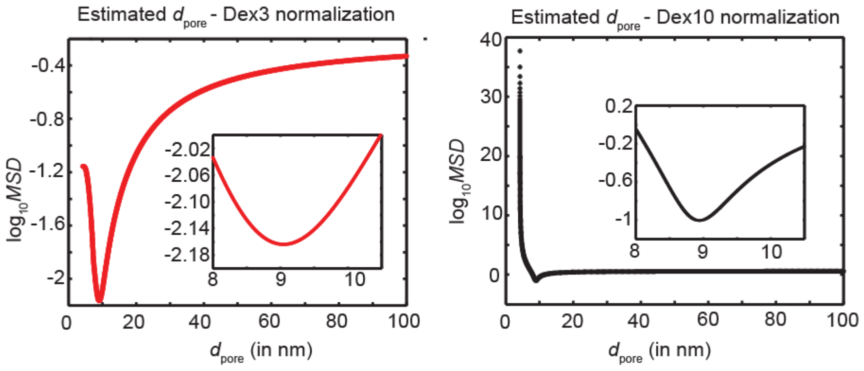

Figure Legend: Determination of the best fit average d_{pore} for different nasal regions obtained by minimizing the mean-of-the-squared-differences (*MSD*) between *X* and *Y* values (equation (10)) for a particular tracer normalization in a region (supplementary section 2).

References:

1 Bungay & Brenner. The motion of a closely-fitting sphere in a fluid filled tube. *Int. J. Multiphase Flow.* **1**, 25-56 (1973).

2 Pappenheimer, J. R., Renkin, E. M. & Borrero, L. M. Filtration, diffusion and molecular sieving through peripheral capillary membranes; a contribution to the pore theory of capillary permeability. *Am J Physiol* **167**, 13-46 (1951).

3 Grotte, G. Passage of dextran molecules across the blood-lymph barrier. *Acta Chir Scand Suppl* **211**, 1-84 (1956).

4 Wasserman, K. & Mayerson, H. S. Dynamics of lymph and plasma protein exchange. *Cardiologia* **21**, 296-307 (1952).

5 Deen, W. M. Hindered transport of large molecules in liquid-filled pores. *AiChe Journal* **33**, 1409-1425 (1987).

6 Sarin, H. Physiologic upper limits of pore size of different blood capillary types and another perspective on the dual pore theory of microvascular permeability. *J Angiogenes Res* **2**, 14, doi:10.1186/2040-2384-2-14 (2010).
